# The polymorphisms of LCR, E6, and E7 of HPV-58 isolates in Yunnan, Southwest China

**DOI:** 10.1186/s12985-018-0986-7

**Published:** 2018-04-25

**Authors:** Juemin Xi, Junying Chen, Miaoling Xu, Hongying Yang, Songjiao Wen, Yue Pan, Xiaodan Wang, Chao Ye, Lijuan Qiu, Qiangming Sun

**Affiliations:** 1 Institute of Medical Biology, Chinese Academy of Medical Sciences, and Peking Union Medical College, Kunming, 650118 People’s Republic of China; 2 Yunnan Key Laboratory of Vaccine Research and Development on Severe Infectious Diseases, Kunming, 650118 People’s Republic of China; 3 Yunnan Provincial Key Laboratory of Vector-borne Diseases Control and Research, Pu’er, 665000 People’s Republic of China; 4 The First Affiliated Hospital of Kunming Medical University, Kunming, 650032 People’s Republic of China; 5 The Third Affiliated Hospital of Kunming Medical University, Yunnan Provincial Tumor Hospital, Kunming, 650118 People’s Republic of China; 6 Kunming Medical University, Kunming, 650500 People’s Republic of China; 7 Institute of Pediatric Disease Research in Yunnan, the Affiliated Children’s Hospital of Kunming Medical University, Kunming, 650228 People’s Republic of China

**Keywords:** HPV-58, LCR, E6, E7, Polymorphism, Phylogenetic analysis, Selection pressure, MHC binding peptides, Transcription factor binding site

## Abstract

**Backgroud:**

Variations in HPV LCR/E6/E7 have been shown to be associated with the viral persistence and cervical cancer development. So far, there are few reports about the polymorphisms of the HPV-58 LCR/E6/E7 sequences in Southwest China. This study aims to characterize the gene polymorphisms of the HPV-58 LCR/E6/E7 sequences in women of Southwest China, and assess the effects of variations on the immune recognition of viral E6 and E7 antigens.

**Methods:**

Twelve LCR/E6/E7 of the HPV-58 isolates were amplified and sequenced. A neighbor-joining phylogenetic tree was constructed by MEGA 7.0, followed by the secondary structure prediction of the related proteins using PSIPRED v3.3. The selection pressure acting on the HPV-58 E6 and E7 coding regions was estimated by Bayes empirical Bayes analysis of PAML 4.8. Meanwhile, the MHC class-I and II binding peptides were predicted by the ProPred-I server and ProPred server. The transcription factor binding sites in the HPV-58 LCR were analyzed using the JASPAR database.

**Results:**

Twenty nine SNPs (20 in the LCR, 3 in the E6, 6 in the E7) were identified at 27 nucleotide sites across the HPV-58 LCR/E6/E7. From the most variable to the least variable, the nucleotide variations were LCR > E7 > E6. The combinations of all the SNPs resulted in 11 unique sequences, which were clustered into the A lineage (7 belong to A1, 2 belong to A2, and 2 belong to A3). An insertion (TGTCAGTTTCCT) was found between the nucleotide sites 7280 and 7281 in 2 variants, and a deletion (TTTAT) was found between 7429 and 7433 in 1 variant. The most common non-synonymous substitution V77A in the E7 was observed in the sequences encoding the α-helix. 63G in the E7 was determined to be the only one positively selected site in the HPV-58 E6/E7 sequences. Six non-synonymous amino acid substitutions (including S71F and K93 N in the E6, and T20I, G41R, G63S/D, and V77A in the E7) were affecting multiple putative epitopes for both CD4^+^ and CD8^+^ T-cells. In the LCR, C7265G and C7266T were the most variable sites and were the potential binding sites for the transcription factor SOX10.

**Conclusion:**

These results provide an insight into the intrinsic geographical relatedness and biological differences of the HPV-58 variants, and contribute to further research on the HPV-58 epidemiology, carcinogenesis, and therapeutic vaccine development.

**Electronic supplementary material:**

The online version of this article (10.1186/s12985-018-0986-7) contains supplementary material, which is available to authorized users.

## Background

Human papillomavirus type 58 (HPV-58) is one of the high-risk members of the papillomavirus family, which are considered to be associated with cervical cancer [1]. Its prevalence is particularly high in East Asia [2]. Based on the viral genomes, HPV-58 variants are classified into 4 variant lineages (A, B, C and D) and 7 sublineages (A1 A2, A3, B1, B2, D1 and D2) [3, 4]. The genome of HPV-58 is a circular double-stranded DNA with 7.9 kb in length, and is comprised of six early open-reading frames (ORFs) (E1, E2, E4, E5, E6, and E7), two late ORFs (L2 and L1), a small non-coding region between E5 and L2, and the long control region (LCR) [5].

The high-risk HPV oncoproteins E6 and E7 can disturb the transcriptional activity of the tumor suppressor protein p53 and the retinoblastoma tumor suppressor gene product pRB, respectively, and induce their proteolytic degradation. E7 can also interact with p53. They are thought to perturb the cell cycle to provide a cellular milieu to favor the replication of the HPV genome DNA and the production of viruses [6]. These effects induce the malignant transformation and immortalization of the infected cells. The HPV LCR is about 850 bp and contains a variety of the regulatory sites for the viral factors and cellular transcriptional factors, like E2 [7, 8], YY1 [9], and sex determining region Y-box 2 (SOX2) [10]. In addition to being useful for the HPV classification, the variations in the LCR have been implicated in the viral persistence and cervical cancer development [11, 12]. The LCR variants have been shown to differentially regulate the replication of HPV throughout the viral life cycle [13] and the transcriptional activity of E6 and E7 [14].

In recent years, there have been many relevant reports about the E6, E7 and LCR of HPV-16. However, studies of the HPV-58 sequences originating in Southwest China are few, especially for the LCR. It is necessary to further research the nucleotide polymorphisms within the HPV-58 LCR, E6, and E7. Besides, the effects of the variations on the immune system’s ability of recognition of the viral E6 and E7 antigens were little reported and should be evaluated. In this paper, the single nucleotide polymorphisms (SNPs) in the HPV-58 LCR, E6, and E7 in Southwest China, were identified and the phylogeny was described to determine the circulating lineages. To assess the possible association of polymorphisms in the HPV-58 E6, E7 and LCR with the virus infection, replication and propagation, the amino acid changes in the HPV-58 E6 and E7 which affect multiple putative epitopes for both CD4+ and CD8+ T-cells were described, and the binding sites of the transcription factors in the LCR were analyzed. Our results could provide basic data for further studies on the HPV-58 epidemiology, prevention, and therapeutic vaccine development.

## Methods

### Ethical statement

Each participant was informed of the study aims for obtaining informed consent. This study was approved by the Institutional Ethics Committee of Institute of Medical Biology, Chinese Academy of Medical Sciences and Peking Union Medical College (No. 2009ZC187M), and was carried out in line with the Helsinki Declaration.

### Cervical samples

Two thousand one hundred forty six cervical scrapings of gynecological outpatients aging from 22 to 59 were collected from the First Affiliated Hospital of Kunming Medical University in Southwest China in 2015. The routine cytology and HC2 testing were performed. Then each sample was stored in a 1.5 mL microtube at − 80 °C for further study.

### DNA extraction and HPV typing

The DNA of cervical scraped cells was extracted according to the previous publications [15, 16]. The pellet of scraped cells was washed in 1 mL washing buffer containing 50 mM Tris-HCl (pH 8.1), 0.5% Tween 20, and 1 mM EDTA followed by digested in 100 μL buffer containing 50 mM Tris-HCl (pH 8.1), 0.1 mg/mL proteinase K, 0.5% Tween 20 and 1 mM EDTA at 55 °C overnight. After the digested pellet was boiled at 95 °C for 10 min, each supernatant was collected by centrifugation at 13,000 rpm for 5 min, each supernatant was transferred into a 1.5 mL microtube and stored at − 80 °C for amplification.

The nested PCR assay was performed for HPV typing. Firstly, the primer pair MY09 (CGTCCAAAAGGAAACTGAGC) /MY11 (GCACAGGGACATAACAATGG) was used to amplify the extracted DNA. The 20 μL PCR reaction volume contained 1 μL of the extracted DNA, 10 μL 2× Power Taq PCR MasterMix (BioTeke), 2 μM MY09 primer and 2 μM MY11 primer. The reaction condition was 94 °C for 5 min, followed by 38 cycles of 94 °C for 1 min, 55 °C for 1 min, 72 °C for 1 min, and then 72 °C for 10 min. Secondly, the primer pair GP5+ (TTTGTTACTGTGGTAGATACTAC) /GP6+ (GAAAAATAAACTGTAAATCATATTC) was used as previously described [17, 18]. 5 μL of the first-round products, 25 μL 2× Power Taq PCR MasterMix (BioTeke), 0.8 μM GP5+ primer and 0.8 μM GP6+ primer were contained in the 50 μL reaction volume. The following thermocycling was used: 95 °C for 5 min, followed by 38 cycles of 95 °C for 1 min, 50 °C for 1 min, 72 °C for 50 s, and then 72 °C for 10 min. β-globin (forward primer: ACACAACTGTGTTCACTAGC, reverse primer: CAACTTCATCCACGTTCACC) was served to assess the sample quality [19], and the PCR reaction without the template DNA was used as a negative control. After the second-round PCR, the PCR products were examined on the 1% agarose gels. The DNA fragments with 150 bp in length were then sequenced bidirectionally by TSINGKE, China and aligned on NCBI for the HPV typing.

### HPV-58 LCR, E6, and E7 sequencing

According to the HPV-58 prototype D90400.1, two partially overlapping fragments of the HPV-58 LCR were amplified by using two pairs of primers to obtain the entire LCR. Primers used for the amplification of the complete nucleotide sequence of the HPV-58 E6 and E7 were described previously [16]. All the primers (Additional file 1: Table S1) were synthesized by TSINGKE, China.

The E6, E7 and LCR fragments were amplified in 50 μL PCR reaction volumes containing 2 μL of extracted DNA, 25 μL 2× Power Taq PCR MasterMix (BioTeke), 0.4 μM forward primer and 0.4 μM reverse primer. The PCR condition was: 95 °C for 5 min, followed by 40 cycles of 95 °C for 30 s, 55 °C for 45 s, 72 °C for 50 s, and then 72 °C for 10 min. The positive fragments were subjected to the bi-directional DNA sequencing (TSINGKE, China) for further analysis. The sequences obtained in this study were submitted to the NCBI GenBank database and got the accession number: MF741321 to MF741331.

### Variant identification and analysis

To identify the SNPs in the LCR, E6 and E7, the studied HPV-58 LCR/E6/E7 sequences were aligned to the HPV-58 prototype sequence (GenBank: D90400.1) by MEGA (version 7.0) [20] after sequencing.

The secondary structures of the HPV-58 E6 and E7 proteins were predicted by PSIPRED v3.3 (http://bioinf.cs.ucl.ac.uk/psipred/) using the default parameters [21].

The neighbor-joining phylogenetic tree based on the HPV-58 LCR/E6/E7 was constructed by MEGA software (version 7.0) using the Kimura 2-parameter model. The number of bootstrap replications was set at 1000. To construct the phylogenetic branches, the following reference sequences were used: D90400.1 (A1), HQ537752 (A2), HQ537758 (A3), HQ537762 (B1), HQ537764 (B2), HQ537774 (C), HQ537768 (D1) and HQ537770 (D2) [3].

To estimate the selection pressure acting on the HPV-58 E6 and E7 coding regions, the non-synonymous and synonymous nucleotide divergence were calculated by CODEML in PAML 4.8. Using the Bayes empirical Bayes (BEB) analysis, the sites with the posterior probability > 95% were identified as the positively selected sites [22–25].

### MHC binding peptides prediction

The ProPred-I server (http://www.imtech.res.in/raghava/propred1/) and ProPred server (http://www.imtech.res.in/raghava/propred/) were used to predict the major histocompatibility complex (MHC) class-I binding peptides [26] and MHC class-II binding peptides [27], respectively.

### Transcription factor binding sites prediction

To analyze the transcription factor binding sites in the HPV-58 LCR, the JASPAR database (http://jaspar.genereg.net/) [28] was used. The sites for YY1, SRY, CEBPA, SOX9, SOX10, POU2F2, JUN, FOS, FOXP3, ETS1, SPIB, ESR2, HSF1, CREB1, PHOX2A, NFIA, ELK4, SMAD3, IRF2, STAT1, HOXC11, NFKB1, RAX, and VAX1 were included. The relative profile score threshold was set at 85%.

## Results

### HPV-58 prevalence among the collected gynecological outpatient samples

Among the 2146 collected outpatient samples in Southwest China, 248 samples (11.56%, 248/2146) were HPV positive [15]. Following HPV-16 (18.95%, 47/248), HPV-58 was identified as the second most common high-risk type, accounting for 13.71% (34/248) in this population.

### Genomic polymorphisms of HPV-58 LCR/E6/E7

12 entire LCR/E6/E7 sequences were obtained and further analyzed. The small copy number of HPV in some women might be a possible explanation for not all sequences successfully amplified. When aligned to the HPV-58 reference sequences (GenBank: D90400.1), a total of 29 single SNPs were identified in 27 nucleotide sites across the LCR, E6 and E7. The combinations of all these SNPs made for 11 unique LCR/E6/E7 sequences (variant IDs: 1 to 11), of which the variant 2 was represented in 2 samples (Table 1). The maximum pairwise difference of the LCR/E6/E7 sequence between any two variants was approximately 0.8%.

**Table 1:** HPV-58 variants based on the LCR/E6/E7 sequences

		HPV-58 variable sites																											
GenBank Acession	Variant ID	LCR																		E6			E7						Lineage
		7172	7194	7265	7266	7304	7440	7525	7540	7556	7714	7749	7755	7784	7793	7807	24	52	54	307	321	388	632	694	744	760	761	803	
D90400.1		T	G	C	C	A	T	A	A	C	A	C	A	T	A	T	T	C	G	C	C	A	C	G	T	G	G	T	A1
MF741321	1	–	–	–	–	–	G	–	–	–	–	–	–	–	–	–	–	–	A	–	–	–	–	–	–	–	–	–	A1
MF741322	2	–	–	–	–	–	–	–	–	–	–	–	–	–	–	–	–	–	–	–	–	–	–	–	–	–	–	–	A1
MF741323	3	–	–	–	–	–	–	–	–	–	–	–	–	–	G	–	–	–	–	–	–	C	–	–	G	–	–	C	A1
MF741324	4	–	–	–	–	–	–	–	G	–	–	G	–	C	–	–	–	–	–	–	–	–	–	–	–	–	–	–	A1
MF741325	5	–	–	G	T	–	–	–	–	–	G	–	–	–	–	–	–	–	–	T	–	–	–	A	G	–	A	–	A2
MF741326	6	–	–	G	T	–	–	–	–	–	–	–	–	–	G	–	–	–	–	–	T	C	–	–	G	–	–	C	A1
MF741327	7	–	–	–	–	–	–	–	–	–	–	–	–	–	–	–	–	–	–	–	–	C	–	–	–	–	–	–	A1
MF741328	8	C	–	G	T	–	–	–	–	–	G	–	–	–	–	G	–	–	–	T	–	–	–	A	G	–	A	–	A2
MF741329	9	–	C	–	–	G	–	C	–	T	C	–	G	–	C	–	–	T	–	T	–	–	T	–	G	A	–	–	A3
MF741330	10	–	C	–	–	G	–	–	–	–	C	–	G	–	–	–	–	T	–	T	–	–	T	–	G	A	–	–	A3
MF741331	11	–	–	–	–	–	–	–	–	–	–	–	–	–	G	–	G	–	–	–	–	C	–	–	G	–	–	C	A1
AA changes																				C66C	S71F	K93 N	T20I	G41R	T57 T	G63S	G63D	V77A	
Secondary structure																				H					S			H	

The nucleotides matching the reference sequence (GenBank: D90400.1) are marked with a dash (−). The H and S in the row of secondary structure designate “helix” and “sheet”, respectively

In the LCR, 20 SNPs were detected at 18 nucleotide sites (18/794, 2.3% nucleotide variation). C7265G, C7266T and A7793G were the most variable sites and were identified in 27% of the variants (3/11). In addition, an insertion (TGTCAGTTTCCT) was found between the nucleotide sites 7280 and 7281 in 2 variants (variant IDs: 9, 10), and a deletion (TTTAT) was found between the nucleotide sites 7429 and 7433 in 1 variant (variant ID: 2). In the E6, 3 SNPs were detected (3/450, 0.7% nucleotide variation). C307T and A388C were identified in 36% variants (4/11). C321T and A388C resulted in the amino acid changes including S71F and K93 N, respectively. No non-synonymous variations were observed in the E6 sequences encoding the α-helix or the β-sheet. In the E7, 6 SNPs were detected (6/297, 2.0% nucleotide variation), of which T744G was identified in 64% variants (7/11), and 5 SNPs resulted in amino acid changes including T20I, G41R, G63S, G63D and V77A. The non-synonymous variation V77A was detected in the E7 sequences encoding the α-helix. There was no insertion or deletion detected in either E6 or E7 sequences.

### Phylogenetic analysis

A neighbor-joining phylogenetic tree based on the HPV-58 LCR/E6/E7 was inferred from 11 obtained unique variants and 8 reference sequences. As shown in Table 1, all the 11 unique variants representing 12 samples, were clustered into the A lineage, including seven A1 (variant IDs: 1, 2, 3, 4, 6, 7, 11, representing 8 samples), two A2 (variant IDs: 5, 8) and two A3 (variant IDs: 9, 10) sublineages with the proportions of 64%, 18%, and 18%, respectively.

### Selective pressure analysis

The selective pressure analysis results by the Bayes empirical Bayes (BEB) analysis of PAML 4.8 were summarized in Table 2. In the HPV-58 E6 coding sequences, no positive selection was observed. However, in the HPV-58 E7 coding sequences, 63G was determined to be a positively selected site.

**Table 2:** Tests for the positive selected sites in HPV-58 E6 and E7

Regions	Models	lnL	2ΔlnL	Positively selected sites
E6	M1	− 675.08	2.18	NA
	M2	− 673.99		NS
	M7	−675.13	2.28	NA
	M8	−673.99		NS
E7	M1	− 499.87	18.92	NA
	M2	− 490.41		63G**
	M7	−499.84	18.86	NA
	M8	−490.41		63G**

lnL designates the log-likelihood; 2ΔlnL designates 2-fold as the lnL difference between the two models. Two asterisks (**) indicate the posterior probability ≥99% by BEB analysis and to be a positively selected site. NA designates not allowed, and NS designates the sites not reaching the significant level

### Amino acid changes in the MHC binding peptides of the HPV-58 E6 and E7 proteins

To evaluate the impacts of the HPV-58 E6 and E7 sequence polymorphisms on the immune recognition of these antigens, the binding peptides specific for both MHC class-I and class-II were predicted by the ProPred-I and ProPred servers, respectively. The results showed that the non-synonymous substitutions within the HPV-58 E6 and E7 were potentially affecting the multiple epitopes. The results were summarized in Table 3. In the HPV-58 E6, a C to T substitution at the nucleotide site 321 led to the change of residue 71 (S to F) in one isolate, where the MHC class-I binding peptide KVCLRLLSK (E6 64–72) and the MHC class-II binding peptide LRLLSKISE (E6 67–75) were located. The A to C substitution at the nucleotide site 388 resulted in the change of residue 93 (K to N), where the MHC class-I binding peptide TLKKCLNEI (E6 91–99) was located. In the HPV-58 E7, the non-synonymous substitution T20I was affecting the MHC class-I binding peptide HPEPTDLFC (E7 16–24) and the MHC class-II binding peptide LHPEPTDL (E7 15–22). G41R was affecting the MHC class-I binding peptide DEIGLDGPD (E7 35–43) and the MHC class-II binding peptide IGLDGPDGQ (E7 37–45). G63S/D was affecting the MHC classs-I binding peptide CYTCGTTVR (E7 59–67). V77A was affecting the MHC class-I binding peptide TTDVRTLQQ (E7 74–82) and the MHC class-II binding peptide VRTLQQLLM (E7 77–85).

**Table 3:** The putative MHC binding peptides of HPV-58 E6 and E7 proteins

Protein	Position of amino acid	Peptide sequence	MHC binding
E6	64–72	KVCLRLLSK	Class-I
	91–99	TLKKCLNEI	Class-I
	67–75	LRLLSKISE	Class-II
E7	16–24	HPEPTDLFC	Class-I
	35–43	DEIGLDGPD	Class-I
	59–67	CYTCGTTVR	Class-I
	74–82	TTDVRTLQQ	Class-I
	15–22	LHPEPTDL	Class-II
	37–45	IGLDGPDGQ	Class-II
	77–85	VRTLQQLLM	Class-II

### Analysis of the transcription factor binding sites

The potential binding sites for the cellular transcription factors in the HPV-58 LCR variants were investigated by the online tool JASPAR. The results indicated the affected transcription factor binding sites due to some of the nucleotide variations spanning nucleotides 7140 to 109. The variations at the nucleotide sites 7265 and 7266 were suggested to be the binding sites for the transcription factor SOX10, and 7304 and 7714 for SOX9. In addition, the nucleotide sites 7172, 7540, 7556, 7749, 7755, 7793, 7807, and 24 potentially affected the binding sites for the transcription factors FOXA1, SRY, ELK4, FOS, FOXP3, ESR2, CEBPA, and HOXC11, respectively.

## Discussion

A specific geographic and population research on the characterization of each HPV type is important to learn the HPV prevalence, find out the biological evidences of carcinogenesis, and help improving the strategies of vaccine development. So far, the HPV family is comprised of more than 170 different types [29]. They can infect the genital tract, the upper-respiratory tract, or the skin. The high-risk HPV types are considered carcinogenic, such as the types 16, 18, 33 and 58 [1]. In our study, HPV-58, accounting for 13.71% of all the HPV-positive samples, was the second most prevalent high-risk types after HPV-16 in Yunnan, China. The nucleotide substitution C321T in the HPV-58 E6, leading to the amino acid change (S71F) was the novel variation detected in this study when compared with those from 2010 to 2011 [16].

In the HPV-58 genome, each region differs in the sequence diversity. The HPV-58 LCR variants from Yunnan, Southwest China have not been reported yet. Here, the variation of the HPV-58 LCR was showed in a higher ratio than those of the E6 and E7 in Yunnan, Southwest China. From the most variable to the least variable, the nucleotide variations were LCR > E7 > E6 found in 2.3%, 2.0% and 0.7%, respectively, of the studied variants. Additionally, the most common substitutions in the HPV-58 LCR were C7265G, C7266T and A7793G. And the most common non-synonymous substitutions in the HPV-58 E6 and E7 were A388C (K35 N) and T803C (V77A), respectively. V77A in the E7 was observed in the sequences encoding the α-helix. Whether this substitution changed the secondary structure of the E7 protein and then affected the potential ability of the E7 to cause cervical cancer needs to be further studied. Besides, 63G in the HPV-58 E7 coding sequences was determined to be a positively selected site, indicating an adaptive molecular sequence evolution.

The HPV variants co-evolve with the specific populations and their prevalence varies across the geographic regions. In this study, phylogenetic analysis based on the HPV-58 LCR/E6/E7 revealed a high prevalence of the A lineage, among which the A1 sublineage with a proportion of 64% accounted for the majority of HPV-58 variants obtained. No variant belonged to the lineages B, C, or D. Nevertheless, the B lineage was found in Sichuan [30] and Henan, China [4]; D lineage was found in Hong Kong [2] and Liaoning [31], China, suggesting that the HPV-58 variant lineages are differently distributed around China.

Though phylogenetically related, the HPV-58 variants can differ in pathogenicity. The occurrence of C632T and G760A in the HPV-58 E7, which were identified in our study and resulted in the amino acid changes including T20I and G63S, respectively, has been implied to be linked to an increased risk for cervical cancer [32, 33]. The amino acid changes in the HPV-58 E6 and E7 oncoproteins may influence the MHC binding peptides and then have the significant immunological effects on the immune system’s ability of recognition of these viral antigens. The non-synonymous substitution T20I potentially affected the MHC class-I and class-II binding peptides, probably leading to the loss of the CD8+ cytotoxic T cell or CD4+ helper T cell responses, respectively. In addition, G63S/D (a positively selected site) affected the MHC class-I binding peptide, and V77A, the most common non-synonymous substitution in the HPV-58 E7, made the predicted MHC class-II binding peptide VRTLQQLLM (E7, 77–85) disappear. The HPV E6 and E7 act as the promising specific tumor antigens and are available as the therapeutic targets. Adoptive transfer of dendritic cells with the HPV E6 and E7 antigens is able to induce the CD8+ T-cell mediated immune responses [34, 35]. Therefore, it is essential to perform further functional studies concerning the sequence polymorphisms of the HPV-58 E6 and E7 to find out the biological proof for tumorigenicity and to optimize the strategies for the HPV prevention and therapy.

The HPV LCR has been shown to contain kinds of the binding sites for both viral and cellular factors and regulate the replication of HPV and the transcriptional activity of the E6 and E7 through the interactions between the regulatory protein complex and the viral DNA [10, 13, 36]. Certain nucleotide changes in the LCR may influence the tumorigenic process. For example, the variations within the binding sites for the cellular factor YY1 in the LCR were found to increase the viral oncogene expression [9]. In the present study, the variations at the nucleotide sites 7265 and 7266, which were detected as the most variable sites in HPV-58 LCR, were the potential binding sites for SOX10, a transcription factor of the sex determining region Y (SRY)-related high motility group (HMG)-box gene family [37]. Sox10 has been suggested to function as a tumor suppressor in digestive cancers [38] and in prostatic carcinoma [39] since it was silenced or down-regulated in these tumors. A previous study on the HPV-16 LCR showed that SOX2 from the same SRY-HMG-box gene family acted as a transcriptional repressor, down-regulating the expression of the HPV E6 and E7 oncoproteins in a context of squamous cell carcinomas [10].

## Conclusions

This study provides a useful data for understanding the phylogenetic relatedness of the HPV-58 variants, the possible influence of non-synonymous substitutions in the HPV-58 E6/E7 on the T-cell responses, and the impacts of the HPV-58 LCR variations on the bindings of the cellular transcription factors. Further studies of the HPV-58 variant genomes from a large population of the asymptomatic carriers and cervical cancer patients are required to establish the carcinogenesis of HPV-58 and to make improvements to the immunotherapeutic approaches and vaccine development strategies.

## Additional file

Additional file 1: **Table S1.** The PCR primers for validating the variations of HPV-58 LCR, E6 and E7. (DOC 33 kb)

## Abbreviations

HPV-58: Human papillomavirus type 58
LCR: long control region
SNPs: single nucleotide polymorphisms
SOX: sex determining region Y-box
MHC: major histocompatibility complex
the animo acid acronyms A, C, D, E, F, G, H, I, K, L, M, N, P, Q, R, S, T, V and Y: alanine, cysteine, aspartic acid, glutamic acid, phenylalanine, glycine, histidine, isoleucine, lysine, leucine, methionine, asparagine, proline, glutamine, arginine, serine, threonine, valine and tyrosine
