# Supplementary material for: The polymorphisms of LCR, E6, and E7 of HPV-58 isolates in Yunnan, Southwest China
Source: Virol J. 2018 Apr 25;15:76. doi: 10.1186/s12985-018-0986-7 (PMC5918753; doi:10.1186/s12985-018-0986-7)
Supplement: Supplementary file 1 — Table S1. The PCR primers for validating the variations of HPV-58 LCR, E6 and E7. (DOC 33 kb) [file 12985_2018_986_MOESM1_ESM.doc]

**Supplementary Material**

Table S1. The PCR primers for validating the variations of HPV-58 LCR, E6 and E7

| **Primer name** | **Sequence** | **Position** | **Amplicon size (bp)** |
| --- | --- | --- | --- |
| HPV-58 LCR F1 | ttgttgtggtacttacactattttat | 7140-7165 | 477 |
| HPV-58 LCR R1 | ctgcactgcatgtatatatgagtcac | 7616-7591 |
| HPV-58 LCR F2 | ctacaatttaaacaatacagttaa | 7526-7549 | 481 |
| HPV-58 LCR R2 | catgcacagatgtctccaacgc | 182-161 |  |
| HPV-58 E6 F | cgaaaccggtgcatatataaa | 57-77 | 596 |
| HPV-58 E6 R | gctcatagcagaataggtcagtt | 652-630 |
| HPV-58 E7 F | cagtgtgttggagaccccga | 519-538 | 450 |
| HPV-58 E7 R | cctgttcttcgttctattaccgc | 968-946 |

The HPV-58 LCR/E6/E7 primers were designed according to the prototype (GenBank: D90400.1).
